# Potentially reduced fusogenicity of syncytin‐2 in New World monkeys

**DOI:** 10.1002/2211-5463.13555

**Published:** 2023-01-31

**Authors:** Hiyori Shoji, Koichi Kitao, Takayuki Miyazawa, So Nakagawa

**Affiliations:** ^1^ Laboratory of Virus‐Host Coevolution, Institute for Life and Medical Sciences Kyoto University Japan; ^2^ Department of Molecular Life Science Tokai University School of Medicine Isehara Japan

**Keywords:** endogenous retrovirus, envelope protein, New World monkey, placenta, syncytin

## Abstract

Syncytin‐2 is a membrane fusion protein involved in placenta development that is derived from the endogenous retrovirus envelope gene acquired in the common ancestral lineage of New World and Old World monkeys (OWMs). It is known that syncytin‐2 is conserved between apes and OWMs, suggesting its functional importance; however, syncytin‐2 of common marmosets (*Callithrix jacchus*) exhibits lower fusogenic activity than those of humans and OWMs in human cell lines. To obtain insight into the functional diversity of syncytin‐2 genes in primates, we examined the syncytin‐2 gene in New World monkeys (NWMs). We experimentally evaluated the cell fusion ability of syncytin‐2 in humans, *C. jacchus*, and tufted capuchins (*Sapajus apella*). We found that the cell fusion ability of *S. apella* was lower than that of human syncytin‐2. Chimeric syncytin‐2 constructs revealed that the amino acid differences in the surface unit of *S. apella* syncytin‐2 were responsible for the weak cell fusion activity. In addition, genomic sequence analyses of syncytin‐2 revealed that the open reading frames (ORFs) of syncytin‐2 were highly conserved in seven apes and 22 OWMs; however, the syncytin‐2 ORFs of three of 12 NWM species were truncated. Our results suggest that syncytin‐2 in several NWMs may be of less importance than in OWMs and apes, and other syncytin‐like genes may be required for placental development in various NWM species.

## Graphical abstract

Syncytin‐2 induces cell fusion and plays an important role in placentation. Syncytin‐2 was thought to be conserved in simians; however, we found that Syncytin‐2 in *Sapajus apella* shows a lower cell fusion activity than that in humans. Furthermore, genomic sequence analyses revealed that the ORFs of syncytin‐2 are truncated in three species of New World monkeys.

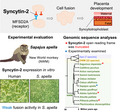

**Abbreviations**
ERV: endogenous retrovirus
ITR: inverted terminal repeat
NWM: New World monkey
ORF: open reading frame
OWM: Old World monkey
RT‐qPCR: reverse‐transcription quantitative PCR
SU: surface unit
TM: transmembrane

Approximately 9% of the human genome consists of endogenous retroviruses (ERVs) [1]. Endogenous retroviruses originate when an exogenous retrovirus infects the host germline; the reverse transcriptase and integrase cause the integration of the entire length or part of the retroviral genome into the host genome. Although ERVs code various functional retrovirus‐derived genes, most have lost their function during the long evolution process; however, some have obtained new functions in the hosts [2]. One example is syncytin genes derived from ERV envelope genes [3, 4]. Syncytin is specifically expressed in the placenta and retains plasma membrane fusion activity as retroviral envelope genes, contributing to the fusion of trophoblast cells in the placenta.

In humans, two syncytin genes—syncytin‐1 and syncytin‐2—have been reported to be involved in placental development, particularly for the cell fusion process of cytotrophoblast cells into a multinucleated layer called syncytiotrophoblast [5]. Syncytin‐1 derived from an ERV‐W envelope was inserted in the ancestor of the Old World monkey (OWM) lineage, but their open reading frames (ORFs) are conserved only in the ape lineage, including humans [6]. Syncytin‐1 protein expresses in placental syncytiotrophoblasts and exhibits cell fusion ability [3, 4]. Meanwhile, syncytin‐2 derived from human endogenous retrovirus (HERV)‐FRD was acquired in the common ancestor of Simiiformes (i.e., simians), including New World monkey (NWM) and OWM lineages, the ORFs of which are reported to be conserved [7]. Human syncytin‐2 expresses primarily in cytotrophoblasts and uses major facilitator superfamily domain‐containing protein 2A (MFSD2A) as a receptor that is expressed predominantly in syncytiotrophoblasts [8]. Therefore, cytotrophoblasts expressing syncytin‐2 fused with syncytiotrophoblasts expressing MFSD2A and transformed into syncytiotrophoblasts [8]. In experiments using BeWo cells and primary human trophoblast cells, cell fusion activity was significantly reduced when the expression of syncytin‐2 was suppressed compared with that of syncytin‐1, suggesting that syncytin‐2 plays a significant role in trophoblast cell fusion in humans [9]. Cell fusion activity of syncytin‐2 was thought to be conserved among simians, including apes, OWMs, and NWMs; however, the syncytin‐2 fusion ability of common marmosets (*Callithrix jacchus*) was observed using feline G355‐5 cells and showed no fusion activity in human 293T and TE671 cells [7]. This observation suggests that the fusion activity of syncytin‐2 genes in NWMs could differ from those of syncytin‐2 in apes and OWMs.

To obtain insights into the functional diversity of syncytin‐2, we investigated the molecular function of syncytin‐2 genes in NWMs. For this purpose, we evaluated the cell fusion ability of syncytin‐2 in the two NWMs, *C. jacchus* and tufted capuchins (*Sapajus apella*, also known as *Cebus apella*), and found that it was lower than that in humans. We also compared the amino acid sequences of syncytin‐2 in 41 species of simians and found they were truncated in some NWMs. Our results suggest that syncytin‐2 might be less important for the cell fusion process during placental development in NWMs, possibly as a result of replacing other syncytin‐like genes.

## Materials and methods

### Cell culture

293T embryonic kidney cells (#RCB2202; Riken BioResource Research Center, Tsukuba, Japan) and G355‐5 feline astrocyte cells (#CRL‐2033; American Type Culture Collection, Manassas, VA, USA) were added to Dulbecco's Modified Eagle's Medium (#5796; Sigma‐Aldrich, Tokyo, Japan) with inactivated fetal bovine serum (Thermo Fisher Scientific, Waltham, MA, USA) and Penicillin–Streptomycin Mixed Solution (#09367‐34; Nacalai Tesque, Kyoto, Japan) and cultured at 37 °C and 5% CO_2_ levels.

### Plasmid

The human syncytin‐2 expression plasmids (phCMV3‐Syn2+400) were used as those previously reported [10]. Genomic DNA was extracted from whole blood samples using a PureLink Genomic DNA Mini Kit (Thermo Fisher Scientific) to construct syncytin‐2 expression plasmids of *C. jacchus* and *S. apella*. Blood samples from *S. apella* (individual number Ca18, male) were collected following a protocol approved by the President of Kyoto University after review by the Institutional Animal Care and Use Committee (permission number: 2012‐017). Blood samples from *C. jacchus* (individual number Cj181, male) were obtained during blood glucose monitoring as part of healthcare, and therefore we did not have a permission number. All individuals were bred and born at the Kyoto University Primate Research Institute.

The syncytin‐2 protein‐coding sequence and 300 bp of the 3′ untranslated region (UTR) were included from the extracted genomic DNA and amplified by polymerase chain reaction (PCR). Amplicons were cloned into EcoRI and BamHI sites of phCMV3 vector (#P003300; Genlantis, San Diego, CA, USA) using NEBuilder HiFi DNA Assembly Cloning Kit (#M5520AA; New England BioLabs Inc., Ipswich, MA, USA). The inserted Syncytin‐2 protein‐coding sequence was confirmed by Sanger sequencing (Fasmac Co., Ltd., Tokyo, Japan) to be identical to the reference genome in RefSeq genes (humans, NM_207582.3; *C. jacchus*, NM_001305096.1; *S. apella*, XM_032249944.1), respectively. To generate human and *S. apella* syncytin‐2 chimeric expression plasmids, human syncytin‐2 expression plasmids were linearized by inverse PCR, and a fragment of *S. apella* syncytin‐2 was inserted using NEBuilder HiFi DNA Assembly Master Mix (New England BioLabs). For cloning of human MFSD2A, we synthesized human cDNA from RNA extracted using an RNeasy Mini Kit (#74104; Qiagen, Hilden, Germany) from 293T cells using a Verso cDNA Synthesis Kit (#AB1453B; Thermo Fisher Scientific). Then, the coding regions of MFSD2A were amplified using PCR. The coding regions of MFSD2A genes of *S. apella* (GenBank ID: XM_032247058.1) were artificially synthesized (Eurofins Genomics K.K., Tokyo, Japan). To generate piggyBac plasmids (pPB‐hsMFSD2A and pPB‐saMFSD2A) expressing human MFSD2A (hsMFSD2A) and *S. apella* MFSD2A (saMFSD2A), the EGFP coding region of the pPB‐EGFP (#VB900088‐2265rnj; VectorBuilder, Chicago, IL, USA) was removed by inverse PCR and replaced with the hsMFSD2A and saMFSD2A sequences using the NEBuilder HiFi DNA Assembly Master Mix (New England BioLabs Inc.). KOD One PCR Master Mix (#KMM‐101; Toyobo, Osaka, Japan) was used for the above PCR. The sequences of the primers are listed in Table S1.

### Cell fusion assay

293T and G355‐5 cells were seeded in 24‐well plates (3 × 10^5^ cells·mL^−1^). The next day, 500 ng of syncytin‐2 expression plasmid was transfected into cells using Avalanche Everyday Transfection Reagent (#EZT‐EVDY‐1; EZ Biosystems, College Park, MD, USA). Cell fusion was observed 7 h after transfection for G355‐5 cells and 20 h after transfection for 293T cells.

### Western blot

Syncytin‐2 expression plasmids were transfected into 293T and G355‐5 cells in the same manner as cell fusion assays. Cells were lysed in RIPA Lysis Buffer (#08714‐04; Nacalai Tesque) from transfection to G355‐5 cells at 7 h and from transfection to 293T cells at 20 h. Cellular suspensions were subjected to glycolysis treatment for 1 h using a PNGase F Kit (New England BioLabs). SDS/PAGE was performed using Mini‐PROTEAN TGX Precast Gels (#4561094; Bio‐Rad Laboratories, Inc., Hercules, CA, USA). Peptides from the gel were transferred to polyvinylidene difluoride membranes, and the monoclonal ANTI‐FLAG M2 antibody (#F3165; Sigma‐Aldrich) was used to detect Flag‐tagged syncytin‐2. Signals were detected using a Super Signal West Femto System (#34095; Thermo Fisher Scientific), and images were obtained using a LAS4000 Mini camera system (Fujifilm, Tokyo, Japan).

### Generation of MFSD2A constitutively expressing cells

To generate 293T cells constantly expressing human and *S. apella* MFSD2A, we utilized the piggyBac transposon system. This system consists of two plasmids. One is an insertable plasmid in which the *MFSD2A* gene and the puromycin‐resistant gene are flanked by inverted terminal repeats (ITRs) (pPB‐hsMFSD2A for human *MFSD2A* and pPB‐saMFSD2A for *S. apella MFSD2A*, respectively). The other plasmid is the hyper PBase expression plasmid (pCAG‐hyPBase) (#VB900088‐2874gzt; VectorBuilder). When these two plasmids were co‐transfected, the hyper PBase protein transfers the region between 5′‐ and 3′‐ITRs into the genomic DNA of the transfected cells. 293T cells were transfected with 0.4 μg of pPB‐hsMFSD2A or pPB‐saMFSD2A and 0.1 μg of pCAG‐hyPBase. At 48 h post‐transfection, cells were selected through puromycin (1 μg·mL^−1^), and the resulting cells served as 293T‐hsMFSD2A and 293T‐saMFSD2A, respectively.

### Quantitative reverse‐transcription PCR

293T and 293T‐hsMFSD2A were seeded in 24‐well plates (3 × 10^5^ cells·mL^−1^), and the RNeasy Mini Kit (#74104; Qiagen) was used to extract RNA. We designed primer sets of the 8th and the 9th exons of human MFSD2A sequences (Table S1), and reverse‐transcription quantitative PCR (RT‐qPCR) reactions were performed using the Power SYBR Green RNA‐to‐CT™ 1‐Step Kit (#4389986; Thermo Fisher Scientific). The reverse transcription (48 °C for 30 min) and denature (95 °C for 10 min) were followed by the denature (95 °C for 10 s) and extension (60 °C for 1 min) with 40 cycles. PCR was carried out using the CFX Connect Real‐Time PCR Detection System (Bio‐Rad Laboratories, Inc.). The MFSD2A expression level of mRNA was normalized by the ACTB expression level. These experiments were performed twice independently.

### Fusion‐dependent luciferase assay

293T cells were seeded in 24‐well plates (4 × 10^5^ cells·mL^−1^). The next day, 500 ng of syncytin‐2 expression plasmids, 500 ng of pT7EMCV‐Luc, and 50 ng of pRL‐TK were transfected into 293T cells. The pT7EMCV‐Luc plasmid expresses firefly luciferase with the internal ribosome entry site of encephalomyocarditis virus in the presence of T7 polymerase. At the same time, another 293T cells were transfected with 500 ng of pCAG‐T7‐pol expressing T7 polymerase. Six hours after transfection, 293T cells were transfected with syncytin‐2 expression plasmid, and pT7EMCV‐Luc and pRL‐TK were co‐cultured with the 293T cells transfected with the T7 polymerase expression plasmid. Then, 24 h after co‐culture, the luciferase activity of the cellular lysates was measured using the Dual‐Luciferase Reporter Assay System (#E1910; Promega, Madison, WI, USA).

### Exploring syncytin‐2 genes in the primate genome

The reference genomes of 65 Euarchonta, including 61 primates (apes, 7 species; OWMs, 22 species; NWMs, 12 species; tarsiers, 1 species; prosimians, 19 species), three treeshrews (*Scandentia*), and one colugo (*Dermoptera*), were downloaded using GenomeSync (https://genomesync.org/; November 21, 2022; Table S2). The coding sequence of the human syncytin‐2 transcript (NM_207582.3) was searched against these reference genomes using tblastn v2.10.0 (*e*‐value < 1E‐50) [11]. The nucleotide sequence of the top match in each genome was extracted with 500 bp of upstream and downstream sequences using the getfasta program in bedtools v2.30.0 [12]. From these sequences, ORFs with more than 500 codons were retrieved using the getorf program in the emboss [13] suite v6.6.0.0, and the amino acid sequences of ORFs were aligned using mafft v7.487 with the L‐INS‐i method [14]. A species tree of the primates used in the analysis was retrieved from TimeTree [15].

### Evolutionary analysis of syncytin‐2 genes

Multiple alignments of syncytin‐2 amino acid sequences of apes (seven species), OWMs (22 species), and NWMs with full coding frames (nine species) were separately generated using mafft v7.487 with the L‐INS‐i method [14]. The resultant alignments were converted to codon alignments. iq‐tree v2.0.3 was used for constructing maximum likelihood trees [16]. Then, the codon alignments and maximum likelihood trees were used for the codeml program in paml v4.8 [17] to conduct the dN/dS analysis based on Nei‐Gojobori method [18]. A single dN/dS ratio for all branches (model = 0) and all sites (NSsites = 0) was estimated in each primate group of apes, OWMs, and NWMs. The significance of the dN/dS ratio was tested by log‐likelihood ratio tests against the null hypothesis (dN/dS = 1).

## Results

### Cell fusion activity of syncytin‐2

**Fig. 1:**
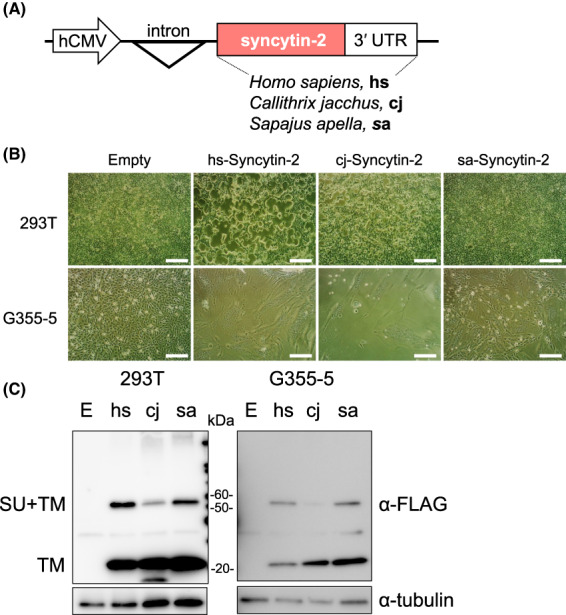
Syncytin‐2 cell fusion assay in humans, common marmosets, and tufted capuchins. (A) Description of syncytin‐2 expression plasmids. hCMV, promoter from human cytomegalovirus. (B) Three syncytin‐2 expression plasmids and empty control plasmids were introduced into 293T and G355‐5 cells, and cell fusion was observed. The shape of the individual cells becomes obscured when cell–cell fusion occurs. A white scale bar indicates 250 μm. (C) A western blot assay was performed using the transfection FLAG‐tagged syncytin‐2 gene. Total cells were harvested when cell fusions were observed (i.e., G355‐5 cells after 7 h and 293T cells after 20 h).

Gene cloning of syncytin‐2 from three simians—humans (*Homo sapiens*), common marmosets (*C. jacchus*), and tufted capuchins (*S. apella*)—was carried out (Fig. 1A). We extended 300 bp at the 3′ ends of the syncytin‐2 ORFs to stabilize their protein expression [10]. The constructed syncytin‐2 expression plasmid was introduced into 293T and G355‐5 cells. By comparing the cell fusion activity of syncytin‐2 among three simians, we revealed that the cell fusion of *S. apella* syncytin‐2 was weaker in both 293T and G355‐5 cells (Fig. 1B). It is known that G355‐5 cells are fusion‐prone cells and they are widely used for cell fusion assays [7]; however, syncytin‐2 of *S. apella* did not clearly exhibit G355‐5 cell fusions. To examine whether the differences in cell fusion efficiency were simply dependent on the expression level of the protein, we conducted a western blot assay with FLAG to the C‐terminus of syncytin‐2. In 293T and G355‐5 cells, the protein expression level of *C. jacchus* Syncytin‐2 was lower than those of *S. apella* and human Syncytin‐2 (Fig. 1C). Thus, the lower cell fusion capacity of syncytin‐2 of *S. apella* may not be due to the lower expression level of the protein.

### Cell‐fusion activity and MFSD2A expression

**Fig. 2:**
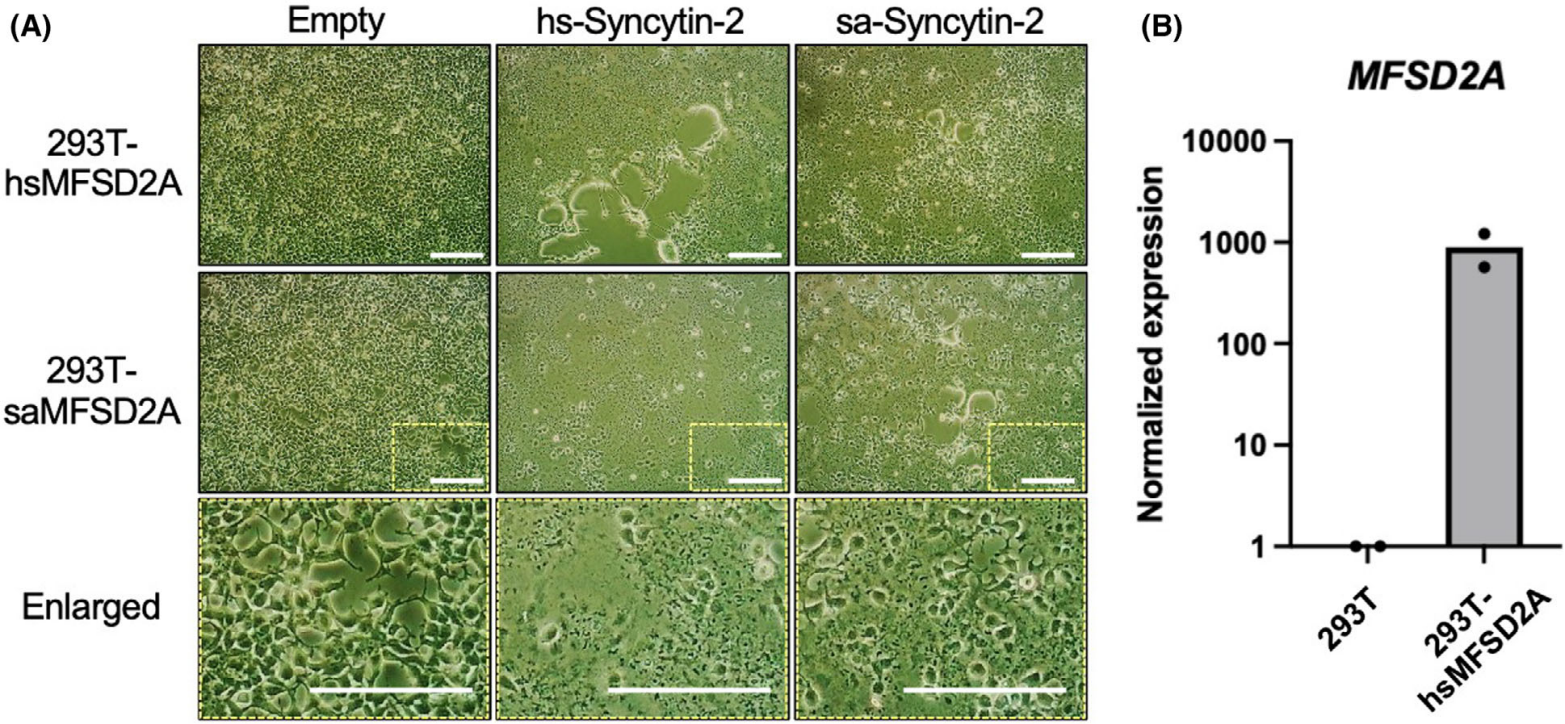
Comparison of cell fusion potential of syncytin‐2 in MFSD2A constitutively expressed 293T. (A) Syncytin‐2 plasmids and empty plasmids were inserted into 293T cells that constitutively express human and *Sapajus apella* MFSD2A (293T‐hsMFSD2A and 293T‐saMFSD2A, respectively). The shape of the individual cells becomes obscured when cell–cell fusion occurs. Also, as the fusion progresses, the fused cells become balloon‐shaped and detach from the bottom of the plate. A white scale bar indicates 250 μm. (B) MFSD2A mRNA expression levels in 293T and 293T‐hsMFSD2A were examined. The expression level of mRNA (log scale) was normalized by actin‐β.

Syncytin‐2 causes cell fusions by binding to cell surface‐expressed MFSD2A [8]. Syncytin‐2 of *S. apella* cannot use human MFSD2A but can interact with MFSD2A of *S. apella*. To confirm this possibility, we generated 293T cells constitutively expressing human and *S. apella* MFSD2A (named 293T‐hsMFSD2A and 293T‐saMFSD2A, respectively). Using these cells, we compared the cell fusion activity of the human and *S. apella* syncytin‐2 genes (Fig. 2A). The results revealed that 293T‐saMFSD2A interacts with both human and *S. apella* syncytin‐2, as indicated by the fusion cells. Interestingly, syncytin‐2 of *S. apella* can cause cell fusion in 293T‐hsMFSD2A as well, which is contrary to the result of the wild‐type 293T cells (Fig. 1B). We assumed that the differences in the expression level of MFSD2A could be responsible for the differences in cell fusion activity. To confirm this, we performed RT‐qPCR to quantify the expression level of MFSD2A. 293T‐hsMFSD2A expressed approximately 1000‐fold more MFSD2A than the wild‐type 293T cells (Fig. 2B). Therefore, it was speculated that the high expression level of the human MFSD2A gene interacting with *S. apella* syncytin‐2 results in cell fusion even if their interaction was weak. These findings suggest that the membrane fusion efficiency of syncytin‐2 of *S. apella* via interaction with MFSD2A is lower than that of human syncytin‐2.

### Identification of regions affecting the fusion ability of syncytin‐2

**Fig. 3:**
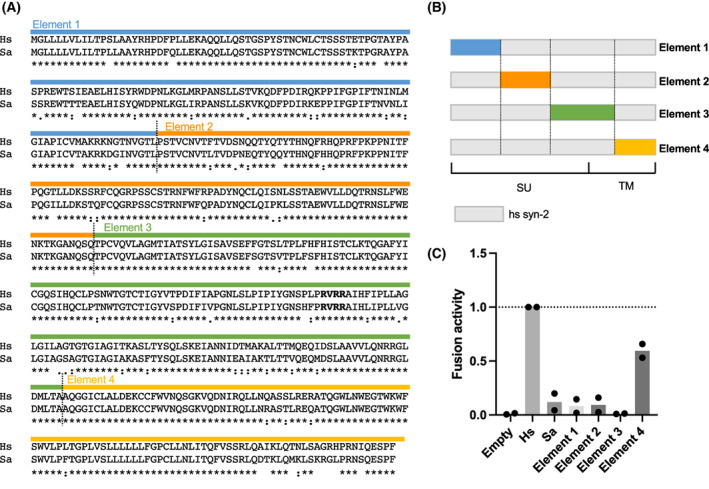
Assessment of the cell fusion capability of syncytin‐2 in humans and tufted capuchins. (A) Amino acid sequence alignment of human syncytin‐2 and *Sapajus apella* syncytin‐2. Syncytin‐2 is cleaved by furin at the furin cleavage site (i.e., RVRR amino acids) shown in bold and divided into SU (surface unit) and TM (transmembrane). (B) Illustration of chimeric syncytin‐2. (C) Comparing the cell fusion ability of chimeric syncytin‐2.

To elucidate the amino acid sequence responsible for the difference in cell fusion ability between human and *S. apella* syncytin‐2 genes, we generated human and *S. apella* chimeric syncytin‐2 (Fig. 3A,B) and compared their cell fusion ability (Fig. 3C). Based on the amino acid sequence alignment of human and *S. apella* syncytin‐2, we split syncytin‐2 into four elements and generated a chimeric syncytin‐2 in which human syncytin‐2 was partially replaced with *S. apella* syncytin‐2 (Fig. 3A,B). The fusion activity of the chimeric syncytin‐2 of human/*S. apella* was evaluated using fusion‐dependent luciferase assay by co‐transfection of a plasmid expressing luciferase luminescent enzyme and substrate into 293T cells (see Materials and methods for details). Cell fusion ability was assessed by cell fusion‐dependent luciferase luminescence (Fig. 3C). Elements 1, 2, and 3, in which a syncytin‐2 element was introduced into the surface unit (SU) region, showed less than 0.1‐fold values of luciferase luminescence, which were similar to those of wild‐type *S. apella* syncytin‐2. By contrast, the luminescence value of Element 4, in which the transmembrane (TM) region was modified, was approximately 0.6‐fold compared with that of wild‐type human syncytin‐2. These results suggest that the reduced cell fusion ability of *S. apella* syncytin‐2 is mainly due to its SU region.

### Truncated syncytin‐2 ORFs in several New World monkeys

**Fig. 4:**
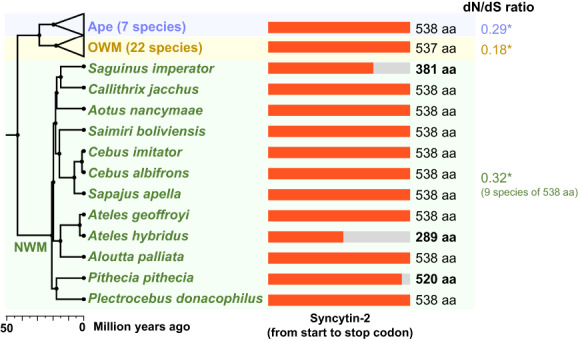
Conservation of syncytin‐2 ORFs in NWMs. Syncytin‐2 genes were obtained from 41 genomes of apes (7 species), OWMs (22 species), and NWMs (12 species). Three NWMs contain stop codons in the syncytin‐2 ORF. The phylogeny and divergence time of each species were obtained from the TimeTree database [15]. The dN/dS ratios are shown on the right. Data were analyzed by log‐likelihood ratio test (*P < 0.05).

The lower cell fusion activity of syncytin‐2 in *S. apella* suggests that the physiological importance of syncytin‐2 in the placenta development of NWMs may be lower than that in apes. To address this hypothesis, we examined the evolutionary conservation of syncytin‐2 obtained from the genomes of 61 primates (apes, 7 species; OWMs, 22 species; NWMs, 12 species; prosimians, 19 species; 1 tarsier species), 3 treeshrews (*Scandentia*), and 1 colugo (*Dermoptera*) (Table S2). We found that all of the apes or OWMs analyzed in this study had 538 amino acid or 537 amino acid lengths of conserved ORFs of syncytin‐2, respectively (Fig. 4). Syncytin‐2 was found in all of the NWM genomes analyzed in this study; however, three species, emperor tamarins (*Saguinus imperator*), brown spider monkeys (*Ateles hybridus*), and white‐faced sakis (*Pithecia pithecia*), had shortened *syncytin‐2* ORFs (Fig. 4). In *S. imperator*, one base insertion occurred in the syncytin‐2 ORF, resulting in a shortened ORF. In *A. hybridus*, one two‐base insertion and two one‐base deletions occurred. In *P. pithecia*, TM regions of syncytin‐2 were shortened by 18 amino acids by the CAG‐to‐TAG mutation.

For the nine NWM *syncytin‐2* sequences remaining full‐length ORFs, we estimated the nonsynonymous and synonymous substitution ratio (dN/dS ratio) to test the hypothesis that purifying selection of syncytin‐2 is more relaxed in NWMs than in apes and OWMs. As a result, the same level of purifying selections was detected in NWMs as in apes and OWMs (Fig. 4; Table S3). The other outgroup primate species (i.e., 19 prosimians and 1 tarsier) do not contain syncytin‐2 sequences in their genomes (Table S2). These findings indicate that the syncytin‐2 inserted in the ancestor of the NWM lineage has not efficiently contributed to the cell–cell fusion process of placental development in several NWMs.

## Discussion

Syncytin‐2 has been considered highly conserved in simians as a fusion gene responsible for placental development [7]. However, our experimental results suggest that syncytin‐2 of tufted capuchins (*S. apella*) showed a lower ability to fuse cells through the interaction between syncytin‐2 and MFSD2A (Figs 1 and 2). This observation was consistent with the cell fusion assay of human 293T cells using the common marmoset (*C. jacchus*) syncytin‐2 [7]. Furthermore, although the amino acid length of syncytin‐2 in *S. apella* and *C. jacchus* is the same as that of humans, three NWMs do not contain intact syncytin‐2 ORFs (Fig. 4). While these results suggest the potential reduction of the Syncytin‐2 fusogenicity in NWMs, intact syncytin‐2 genes of NWMs were under purifying selection (Fig. 4; Table S3). The same observation was reported in *envV2*, which was the envelope gene of ERV‐V inserted into the ancestor of simians [19]. The *envV2* genes were conserved and under purifying selection in simians. However, their fusogenic activity in some NWMs and apes was lost, while those in OWMs, common marmosets, and gibbons are still intact [19]. Together, previous and our studies imply the dynamic birth‐and‐death evolution in fusogenic activity of envelope‐derived genes that are involved in placental development.

We previously proposed the “baton pass” hypothesis for the dynamic evolution of syncytin genes in mammalian genomes [20]. Since various ERVs integrated into mammalian genomes multiple times, newly acquired envelope genes derived from ERVs could have replaced the fusogenic genes previously responsible for cell fusion, the process of which was called the “baton pass” [20]. This hypothesis is based on the observation of ruminant species. In bovines, two syncytin‐like genes, fermatrin‐1 [21] and syncytin‐Rum1 [22], were involved in the cell fusion process during placenta development, and the newly acquired fematrin‐1 showed a more vital ability to fuse cells [21]. Such observations have led to the proposition of the baton pass hypothesis in which the function of a preexisting syncytin‐like gene was transferred to a newly incorporated envelope gene, thus facilitating the evolution of placental morphology [20, 23]. Lavialle et al. also hypothesized that envelope genes involved in placental development have been subsequently replaced in the diverse lineages emerging during the mammalian radiation on successive and independent germline infections by new retroviruses and co‐optation of their envelope genes during the evolution of Theria [24].

The interspecies differences in cell fusion capacity of the syncytin‐2 gene in NWMs revealed in this study support the baton pass hypothesis, as the reduced fusion capacity or loss of the syncytin‐2 gene in some NWMs may facilitate, or result from, the replacement process. It is also considered that syncytin‐like genes derived from envelope genes of ERV have species specificity and redundancy, which may have contributed to the variability in placental morphology in mammalian species [25]. Therefore, the lower placental invasiveness in common marmosets than in apes [26] may be related to the poor cell fusion capacity of syncytin‐2 in NWMs.

In conclusion, this study shows the poor cellular fusion potential of syncytin‐2 in common marmosets and tufted capuchins. Furthermore, although syncytin‐2 ORFs are conserved in apes and OWMs, they are truncated in several NWMs. These findings provide a perspective that, in addition to the divergence of syncytin‐like genes that differ among mammalian lineages, functional fluctuations of identical syncytin‐like genes are also critical to the understanding of placental evolution.

## Conflict of interest

The authors declare no conflict of interest.

## Author contributions

KK, TM, and SN designed the research. HS and KK performed experiments. HS, KK, TM, and SN analyzed the data. HS and KK drafted the initial manuscript. KK, TM, and SN wrote the paper.

## Supporting information


**Table S1.** Primer list.
**Table S2.** Syncytin‐2 search in the 65 Euarchonta genomes.
**Table S3.** Pairwise dN/dS ratios of syncytin‐2.

## Data Availability

All relevant data are contained in the Supporting Information.
